# Graph metric learning quantifies morphological differences between two genotypes of shoot apical meristem cells in *Arabidopsis*

**DOI:** 10.1093/insilicoplants/diad001

**Published:** 2023-01-30

**Authors:** Cory Braker Scott, Eric Mjolsness, Diane Oyen, Chie Kodera, Magalie Uyttewaal, David Bouchez

**Affiliations:** 1 Department of Mathematics and Computer Science, Colorado College, Colorado Springs, CO 80903, USA; 2 Department of Computer Science, University of California Irvine, Irvine, CA 92697, USA; 3 Los Alamos National Laboratory, Los Alamos, NM 87544, USA; 4 Université Paris-Saclay, INRAE, AgroParisTech, Institut Jean-Pierre Bourgin (IJPB), 78000 Versailles, France; 5 CryoCapCell, Inserm U1195, Université Paris Saclay, 94270 Le Kremlin-Bicêtre, France

**Keywords:** Cell morphology, graph metrics, morphodynamics, neural networks, spectral graph theory

## Abstract

We present a method for learning ‘spectrally descriptive’ edge weights for graphs. We generalize a previously known distance measure on graphs (graph diffusion distance [GDD]), thereby allowing it to be tuned to minimize an arbitrary loss function. Because all steps involved in calculating this modified GDD are differentiable, we demonstrate that it is possible for a small neural network model to learn edge weights which minimize loss. We apply this method to discriminate between graphs constructed from shoot apical meristem images of two genotypes of *Arabidopsis thaliana* specimens: wild-type and *trm678* triple mutants with cell division phenotype. Training edge weights and kernel parameters with contrastive loss produce a learned distance metric with large margins between these graph categories. We demonstrate this by showing improved performance of a simple k-nearest-neighbour classifier on the learned distance matrix. We also demonstrate a further application of this method to biological image analysis. Once trained, we use our model to compute the distance between the biological graphs and a set of graphs output by a cell division simulator. Comparing simulated cell division graphs to biological ones allows us to identify simulation parameter regimes which characterize mutant versus wild-type *Arabidopsis* cells. We find that *trm678* mutant cells are characterized by increased randomness of division planes and decreased ability to avoid previous vertices between cell walls.

## 1. Introduction

Plant organogenesis results in stereotypic patterning of cell shapes and cell wall networks. The *Arabidopsis* shoot apical meristem (SAM) is an organogenic, self-rejuvenating tissue that produces the primordia of aerial organs such as leaves and flowers. The morphogenetic activity of the SAM requires tight spatio-temporal control of the timing and orientation of cell divisions, as well as the direction and rate of cell growth. Cell wall networks resulting from SAM activity have been previously described by cell geometry approaches (Hamant *et al.* 2008; Stamm *et al.* 2017; Montenegro-Johnson *et al.* 2019). These methods can quantify local morphology and can be used to calculate tissue-scale topological statistics. However, previous graph metric approaches to quantifying SAM networks have been limited to using topological features to compare cell morphology. Here, we introduce a method for direct comparison of cell networks, using a trained distance metric that incorporates both topological and geometric information. The main scientific goal of the present work is to show that this type of distance metric, once learned, is able to (i) accurately classify plant cells by type, and (ii) can be interpreted to generate insight into the morphological properties of different cell genotypes. Our numerical experiments demonstrate that incorporating geometric information into a learned distance metric outperforms cell-type classifiers operating on topological features, as in Sahlin *et al.* (2009).

Graph diffusion distance (GDD) is a measure of similarity between graphs originally introduced by Hammond *et al*. (2013) and substantially generalized and scaled up by Scott *et al*. (Scott and Mjolsness 2021). This metric measures the similarity of two graphs by comparing their respective spectra (the eigenvalues of the graph Laplacian) as they evolve under graph diffusion. However, it is well-known that there exist pairs of *cospectral* graphs which are not isomorphic but have identical spectra. Furthermore, because even a small change to entries of a matrix may change its eigenvalues, another limitation of GDD is that it is sensitive to small changes in the topology of the graph (as well as small variations in edge weights). Finally, since GDD does not make use of edge or node attributes, it cannot distinguish between two different signals on the same source graph, diminishing its applicability in data science. In this work, we introduce several generalizations to GDD which resolve these issues and make it a powerful machine learning tool for data sets of graphs derived from biological microscope images.

### 1.1. Contributions

The first contribution of this paper is the development of a differentiable version of the GDD calculation. This allows us to backpropagate error through the GDD calculation to the edge weights of the graph, which in turn allows us to learn discriminative edge weights with a neural network. We apply this method to a data set of graphs extracted from confocal microscope images of the L1 cell layer of SAMs of *Arabidopsis thaliana*. Our novel distance calculation method allows us to compare our real graphs with synthetic examples generated via model-derived simulation. On this basis we conclude that a specific genotype of mutant *Arabidopsis* is characterized by increased randomness in the direction of cell division and decreased ability to avoid previous vertices between cell walls.

## 2. GRAPH DIFFUSION DISTANCE

**Figure 1.**
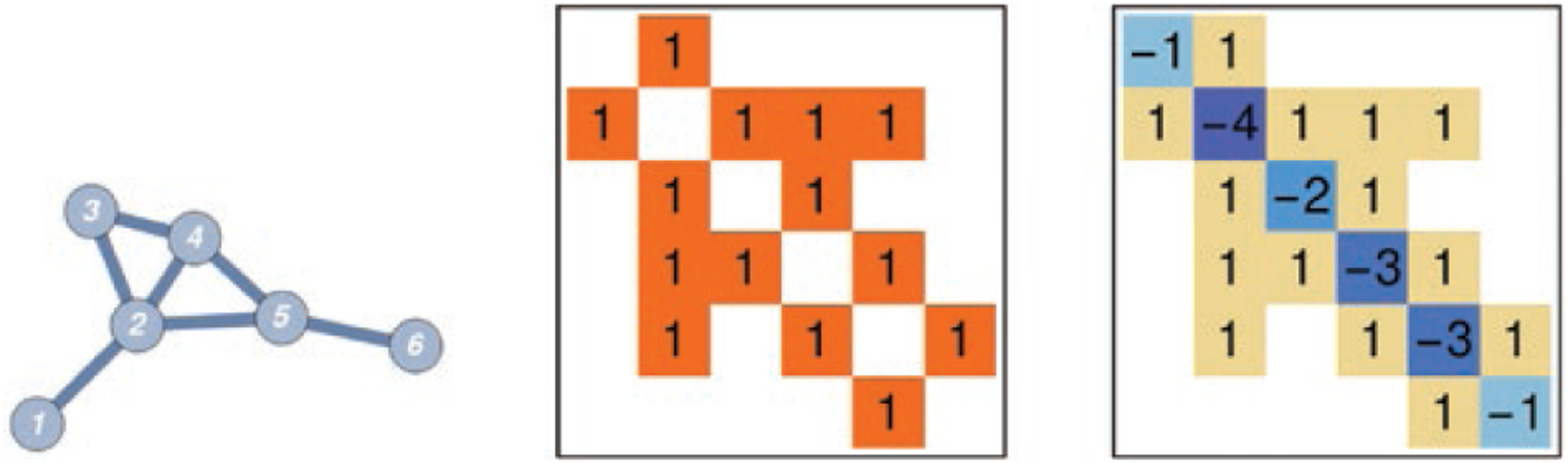
From left to right: a small graph, its adjacency matrix and its graph Laplacian.

We use the definition of GDD first given by Hammond *et al*. and later expanded (to cover differently sized graphs) by Scott *et al*. This graph comparison metric makes use of the eigenvalues of each graph’s Laplacian matrix, which we define as follows: Let $w_{ij}$ be the weight of the edge between the two vertices $v_{i}$ and $v_{j}$. Then, the graph Laplacian is a matrix L=A-D, where A is the (weighted) adjacency matrix of the graph with $w_{ij}$ as the (i,j) th entry. D is a diagonal matrix where the ith entry on the diagonal is given by $\sum_{k}\;w_{ik}$. See Fig. 1 for an example of a graph and its Laplacian. Given two graphs $G_{1}$ and $G_{2}$, let $L_{1}$ and $L_{2}$ be their respective Laplacian matrices. Furthermore, let $L_{i}=U_{i}\Lambda_{i}U_{i}^{T}$ be the diagonalizations of each Laplacian, so that $\Lambda_{i}$ is a diagonal matrix whose jth diagonal entry, $\lambda_{j}^{\left(i\right)}$, is the j th eigenvalue of $L_{i}$. If L is a graph Laplacian, then the matrix $e^{tL}$ (called the *diffusion kernel* of the graph) describes heat flow between vertices of that graph: the (i,j)th entry of the *diffusion kernel* describes the amount of heat that has flowed from node i to node j after t time has passed. Then the GDD between two graphs of the same size is given by comparing the eigenvalues of their *diffusion kernel*s:

(1) $$\begin{array}{rr} D\left(G_{1},G_{2}\right) & \;=\underset{t}{\text{sup}}∥e^{-tL_{1}}-e^{-tL_{2}}∥F=\underset{t}{\text{sup}}∥e^{-t\Lambda_{1}}-e^{-t\Lambda_{2}}∥F\\ & \;=\underset{t}{\text{sup}}\sqrt{\overset{n}{\underset{j=1}{\sum }}{\left(e^{-t\lambda_{j}^{\left(1\right)}}-e^{-t\lambda_{j}^{\left(2\right)}}\right)}^{2}} \end{array}.$$

**Figure 2.**
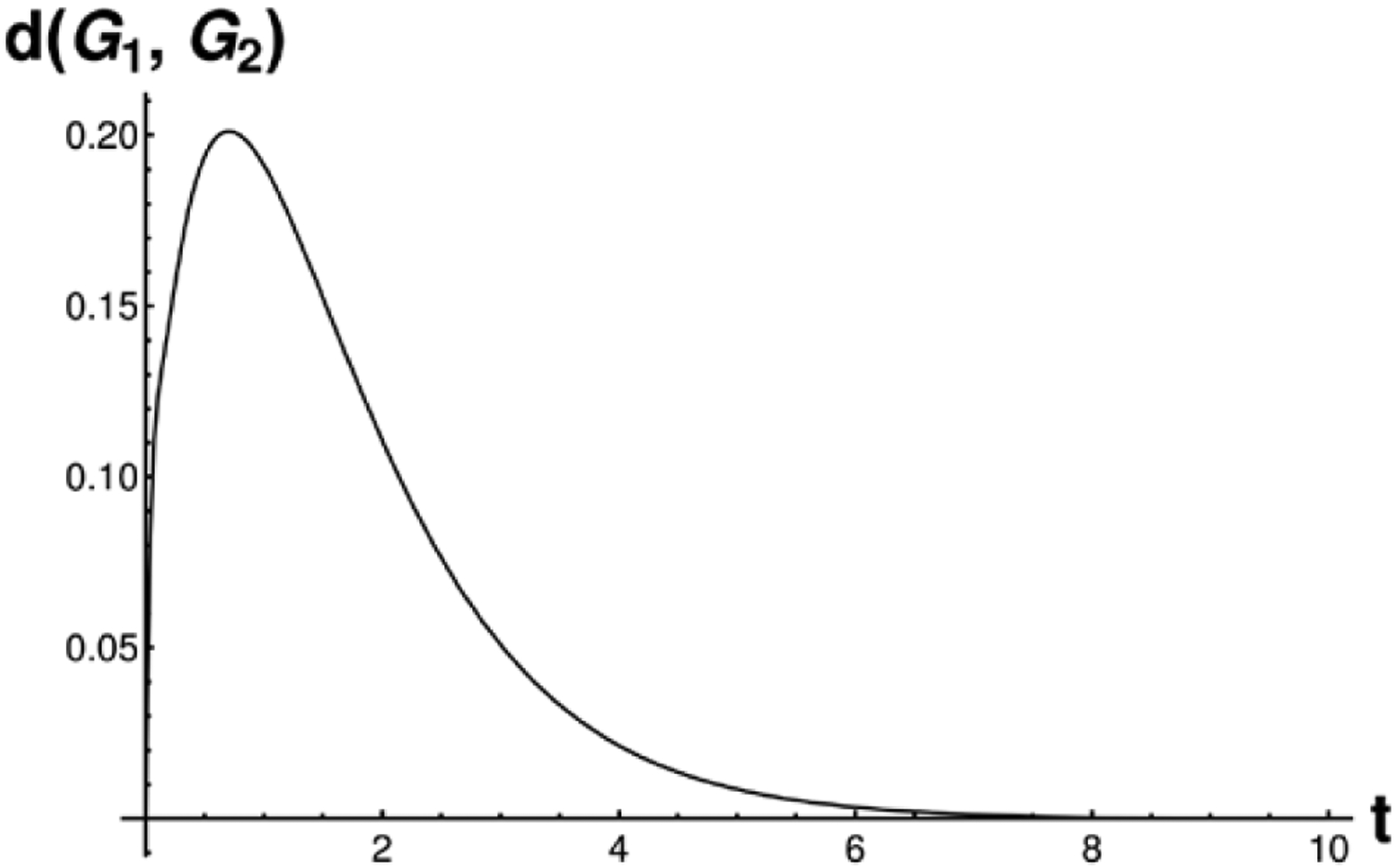
This figure shows the result of evaluating the norm in Equation (1) with eigenvalues of two random graphs on 32 nodes, for varying values of t. This demonstrates why we take the diffusion distance to be the peak of this norm; since at early and late times the difference between the eigenvalues of the two *diffusion kernel*s vanishes.

This equation compares the eigenvalues using the *Frobenius norm*, also known as the element-wise L2 norm. The maximization over t is because we want to compare the two kernels at the most informative time: at both very small and very large values of t, all graphs look identical (since, respectively, either no heat has diffused, or heat has spread evenly everywhere). See Fig. 2 for an example GDD calculation, evaluated at multiple values of t. The simplification in Equation (1) (which we will not detail here) relies on the rotation invariance of the Frobenius matrix norm $∥\cdot {∥}_{F}$ and the Taylor expansion of the exponential map. It is clear that this distance measure requires the two graphs to be the same size, since otherwise this matrix difference is not defined.

The generalization to different-sized graphs given by Scott *et al*. could also be modified in the way we discuss in Section 3, but we do not consider this version of GDD in this paper. Given two graphs *G*_1_,*G*_2_ of differing sizes $n_{1}<n_{2}$, we can define GDD similarly to Equation (1):

(2) $$\begin{array}{rr} D\left(G_{1},G_{2}\right) & \;=\underset{t>0}{\text{sup}}\;\underset{\alpha >0}{\text{inf}}\;\underset{P|𝒞\left(P\right)}{\text{inf}}{\left\|Pe^{\frac{-t}{\sqrt{\alpha }}L_{1}}-e^{-t\sqrt{\alpha }L_{2}}P\right\|}_{F}\\ & \;=\underset{t>0}{\text{sup}}\;\underset{\alpha >0}{\text{inf}}\;\underset{\tilde{P}|𝒞\left(\tilde{P}\right)}{\text{inf}}{\left\|\tilde{P}e^{\frac{-t}{\sqrt{\alpha }}{\text{Λ}}_{1}}-e^{-t\sqrt{\alpha }{\text{Λ}}_{2}}\tilde{P}\right\|}_{F} \end{array}.$$

In Equation (2), α is a time-dilation factor which dilates the passage of time in one graph with respect to the other. Dilating time in the two graphs with α allows us to fairly compare graphs that have radically different sizes. As an example, diffusion on a fine square grid and a coarse square grid look very similar, but with differing timescales. P is a rectangular matrix which is optimized according to some set of constraints 𝒞. In the cited paper by Scott *et al.*, the constraint 𝒞 is taken to be orthogonality: $P^{T}P=I$. The reason for requiring P to be an orthogonal matrix is that optimization over this class of matrices is invariant to rotation of the two coordinate systems of each of the two graphs. Our optimization is then comparing the discrepancy between the two *diffusion kernel*s, regardless of their individual coordinate systems. $\tilde{P}=U_{2}^{T}PU_{1}$ is a change of basis from graph-space to eigenspace, allowing us to again represent the equation for varying-size GDD as a comparison between lists of eigenvalues.

### 2.1. GDD is a differentiable function of t and edge weights

Once all of the eigenvalues $\lambda_{i}$ and orthonormal eigenvectors $v_{i}\;$ (of a matrix L) are computed, we may backpropagate through the eigendecomposition as described in Nelson (1976) and Andrew *et al.* (1993). If our edge weights *A * (and therefore the values in the Laplacian matrix L) are parameterized by some parameter value θ, and our loss function ℒ is dependent on the eigenvalues of L, then we can collect the gradient $\frac{\partial ℒ}{\partial \theta }$ as:

(3) $$\frac{\partial ℒ}{\partial \theta }=\underset{k}{\sum }\left(\frac{\partial ℒ}{\partial \lambda_{k}}\frac{\partial \lambda_{k}}{\partial \theta }\right)=\underset{k}{\sum }\left(\frac{\partial ℒ}{\partial \lambda_{k}}v_{k}^{T}\frac{\partial L}{\partial \theta }v_{k}\right),$$

where $v_{k}$ is the kth unit-length eigenvector of L. This derivation uses the fact that (since the eigenvectors $v_{k}$ are orthonormal)

(4) $$\frac{\partial \lambda_{k}}{\partial \theta }\;=\frac{\partial }{\partial \theta }\left(v_{k}^{T}Lv_{k}\right)=\frac{\partial v_{k}^{T}}{\partial \theta }Lv_{k}+v_{k}^{T}\frac{\partial L}{\partial \theta }v_{k}+v_{k}^{T}L\frac{\partial v_{k}}{\partial \theta }$$

(5) $$=\lambda_{k}\frac{\partial }{\partial \theta }\left(v_{k}^{T}v_{k}\right)+v_{k}^{T}\frac{\partial L}{\partial \theta }v_{k}=0+v_{k}^{T}\frac{\partial L}{\partial \theta }v_{k}$$

If the entries of L are computed as a function of θ using an automatic differentiation package (such as PyTorch; Paszke *et al.* 2019) the gradient matrix $\frac{\partial L}{\partial \theta }$ is already known before eigendecomposition. We note here that for any fixed value of t, all of the operations needed to compute GDD are either simple linear algebra or continuous or both. Therefore, for any loss function ℒ which takes the GDD between two graphs as input, we may optimize ℒ by backpropagation through the calculation of GDD using Equation (3). We note here that although all numerical experiments in this paper use the same-sized version of GDD (Equation (1)), this backpropagation will work for the varying-sized version as well, allowing gradients to be used to adjust any of the inputs to the general GDD equation (Equation (2)).

## 3. LEARNING PARAMETERS FOR *DIFFUSION KERNELS*

In this section, we describe our method for learning edge weights for Laplacian *diffusion kernel*s, beginning with our generalization of GDD to make it trainable, and then introducing a method for learning edge weights.

### 3.1. Diff2Dist: differentiable GDD

We make two main changes to GDD to make it capable of being tuned to specific graph data. First, we replace the real-valued optimization over t with a maximum over an explicit list of t values $t_{1},t_{2},\ldots t_{p}$. This removes the need for an optimization step inside the GDD calculation. We initialize $t_{i}$ to be exponentially distributed in the range $\left[e^{-3},e^{3}\right]$. The t values are updated via gradient descent during training. Second, we re-weight the Frobenius norm in the GDD calculation with a vector of weights $\beta_{j}$ which is the same length as the list of eigenvalues (these weights are normalized to sum to 1). The resulting GDD calculation is then:

(6) $$D\left(G_{1},G_{2}\right)=\underset{t\in t_{1},t_{2},\ldots t_{p}}{\text{max}}\sqrt{\overset{n}{\underset{j=1}{\sum }}\beta_{j}{\left(e^{-t\lambda_{j}^{\left(1\right)}}-e^{-t\lambda_{j}^{\left(2\right)}}\right)}^{2}}.$$

We call this version of GDD *Differentiable Graph Diffusion Distance,* or Diff2Dist. Because this distance calculation is comprised entirely of differentiable components and linear algebra, it may be explicitly included in the computation graph (e.g. in PyTorch) of a machine learning model, without needing to invoke some external optimizer to find the supremum over all t. We note here that although the max() function is not differentiable everywhere, PyTorch has logic for backpropagating through the max() operation. This is accomplished by masking the reverse-mode gradient to only apply to the maximum entry of a given tensor, and breaking ties with the subgradient method—see the PyTorch documentation for more details. $t_{n}$ and $\beta_{j}$ may be tuned by gradient descent or some other optimization algorithm to minimize a loss function which takes $D\left(G_{1},G_{2}\right)$ as input. Tuning the $t_{n}$ values results in a list of values of t for which GDD is most informative for a given data set, while tuning $\beta_{j}$ re-weights GDD to pay most attention to the eigenvalues which are most discriminative. In the experiments in Section 4.2 we demonstrate the efficacy of tuning these parameters using contrastive loss.

### 3.2. Learning edge weighting functions

Here, we note that if graph edge weights are determined by some differentiable function f parameterized by parameters θ, we may still apply all of the machinery of Sections 2.1 and 3.1. A common edge weighting function for graphs embedded in Euclidean space is the *Gaussian Distance Kernel,* $w_{ij}=\text{exp}\left(\frac{-d_{ij}^{2}}{2\sigma^{2}}\right)$, where $d_{ij}$ is the distance between nodes i and j in the embedding. σ is the standard deviation of the distance kernel and can be chosen *a priori* or tuned in the same way as β and t, using a numerical optimization procedure. However, the Gaussian distance kernel, while mathematically well-motivated for embedded graphs, is a somewhat arbitrary choice of edge weight, especially in cases where the edges of our graphs have more complicated edge labels. In cases like the data discussed in this paper, our edge labels are vector-valued, and it is therefore advantageous to replace this hand-picked edge weight with weights chosen by a general function approximator, e.g. an artificial neural network (ANN; Fukushima and Miyake 1982), with inputs defined geometrically. As before, the parameters of this ANN can be tuned using gradients backpropagated through the GDD calculation and eigendecomposition.

## 4. NUMERICAL EXPERIMENTS

### 4.1. Data description

#### 4.1.1 *Arabidopsis* SAM data set.

The species A*. thaliana* is of high interest in plant morphology studies, since it is a standard genetic model organism whose genome was fully sequenced in 1996, relatively early (Kaul *et al*. 2000). Additionally its structure makes it relatively easy to capture images of the aerial stem cell niche with active cell division: the *SAM*. Recent work (Schaefer *et al*. 2017) has found that mutant *Arabidopsis* specimens with a simultaneous loss of function of genes TRM6, TRM7 and TRM8 demonstrate more variance in the placement of new cell walls during cell division. This is thought to be the result of the *trm678* triple mutants having abnormal or absent pre-prophase bands (PPBs) before cell division. The PBB is a microtubule cytoskeleton array which is hypothesized to fine-tune the placement of new cell walls before division (Schaefer *et al*. 2017).

The data set used in this paper was prepared as follows:

1. Two genotypes of *Arabidopsis* (wild-type and *trm678* mutants: mutants with loss of function of all three of TRM6, TRM7 and TRM8) expressing the PDF1::mCITRINE-KA1 reporter (Stanislas *et al.* 2018) were sown and kept in short-day conditions (8h of light, 16 h of dark) for 6 weeks.
2. Plants were transferred to long-day conditions (16 *h* of light, 8h of dark) and kept there until the inflorescence meristem (SAM) had formed. This took 2 weeks for wild-type plants and 3 weeks for *trm678* mutants.
3. The SAM of each plant was then dissected and observed with a confocal microscope, Leica SP8 upright scanning confocal microscope equipped with a water immersion objective (HC FLUOTAR L 25x/0.95 W VISIR).
4. This resulted in 3D stacks of the SAM imaged from above, collected for both types of specimens.
5. Each 3D image was converted to a 2D image showing only the cell wall of the top layer of cells in the SAM. In total we used 20 confocal microscope images (13 from wild-type plants and 7 from *trm678* mutants).
6. Each image was segmented into individual cells, by finding connected components separated by cell walls. This step was performed with the watershed algorithm, as implemented in the Python package scikit-learn (with default parameters).
7. We construct a graph from the resulting segmented image. In this graph, two cells are connected by an edge if they share a boundary and their centres of mass (approximated as the mean coordinate of the corresponding region of pixels) are closer than 100 pixels.
8. Finally, we extracted multiple subgraphs from each of these graphs. Each subgraph consists of a cell and its 63 closest neighbours 63 was chosen so that each of the graphs in our data set had 64 nodes total; we picked this size of graph because it was the largest power of 2 size for which we could fit reasonable batches in memory. Cell neighbourhood selection was limited to the central region of each SAM image, since the primordia (secondary growths surrounding the SAM) are known to have different morphological properties. For each cell neighbourhood, we produce a graph by connecting two cells if and only if their shared boundary is 30 pixels or longer (pixel size: 1 px = 0.1818 μm. For each edge, we save the length of this shared boundary, the angle of the edge from horizontal, and the edge length, as the three geometric attributes input to the ANN that computes graph edge weight.

**Figure 3.**
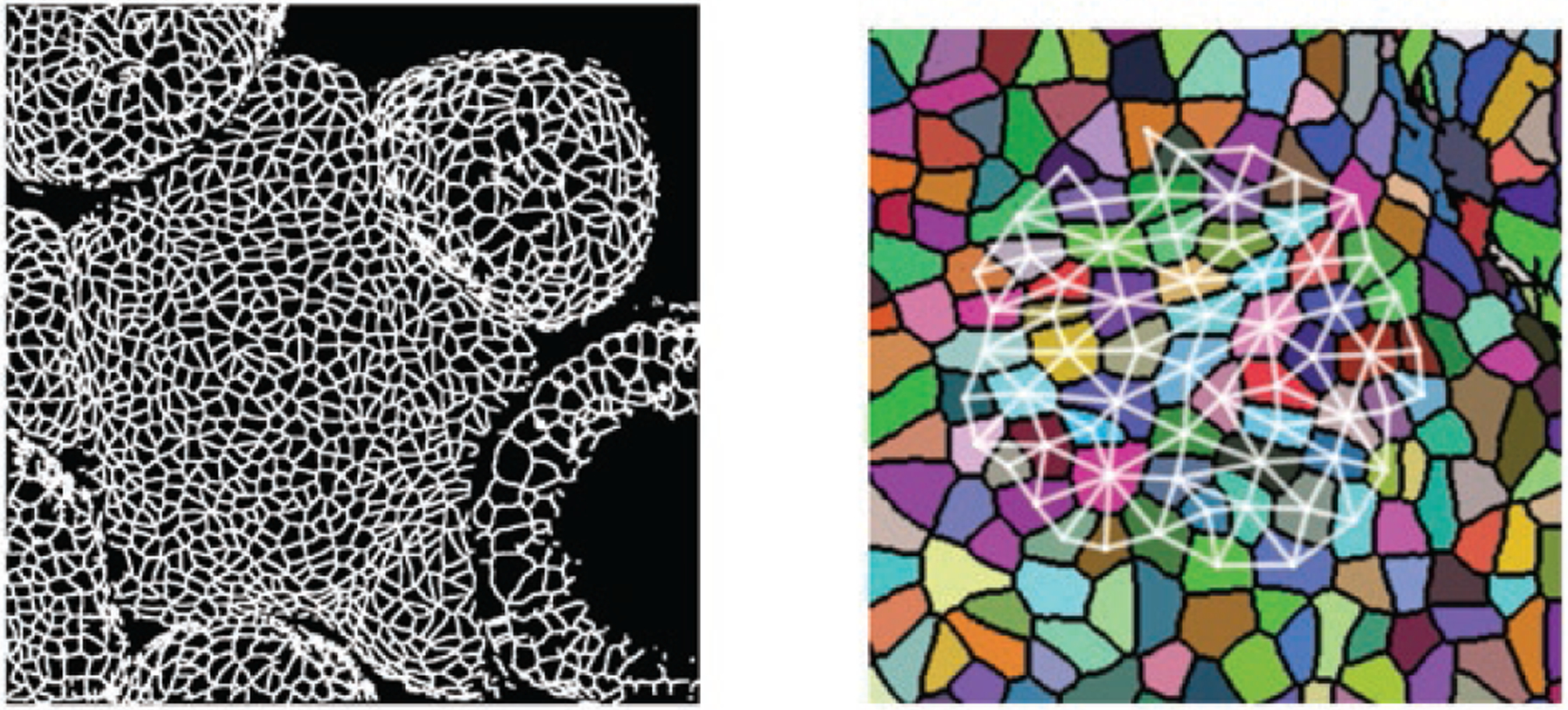
Left: an image of the SAM of a mutant *Arabidopsis* specimen. The original 3D confocal microscope image is here represented as a 2D skeleton. Right: a zoomed-in view of the same specimen, with separate cells false-coloured and an example extracted cell neighbourhood graph overlaid.

We extracted 1200 cell neighbourhoods in this way, resulting in a data set of 600 graphs from each *Arabidopsis* genotype. See Fig. 3 for an example of cell segmentations and extracted graphs. The data set of labelled graphs is available in Code Repository accompanying this paper.

#### 4.1.2 Replum cell data set.

To further validate our proposed approach, we also demonstrate our model’s ability to learn a distance function on a different cell graph data set, also derived from *Arabidopsis* cells. This data set consists of confocal microscope images of the replum of the gynoecium of *Arabidopsis* plants in two varieties: wild-type and mutants with loss of function in all four of the genes TRM1, TRM2, TRM3 and TRM4 (*‘trm1234* mutants’). Cellular descriptions in terms of the orientation of cell divisions and directions of cell growth during *Arabidopsis* gynoecium development are still lacking; however, we took advantage of the organization of the replum epidermis into pseudocells to compare the orientation of cell divisions and the direction of cell growth in the *trm1234* mutants compared to the wild type. We detected cell outlines with the membrane marker PDF1::TdTOMATO-29–1 (adapted from pUBQ::TdTO-MATO-29–1, as in Shapiro *et al.* (2015)). Images of marked cell outlines were processed and turned into graphs with an identical pipeline to the one described in the previous section, with the exception that for this data set we had to take subgraphs of size 32 rather than 64 (since there are fewer cells per image, a 64-cell subgraph would be the entire tissue sample in some cases).

### 4.2. Evaluation of Diff2Dist variants

We test each of the GDD generalizations proposed, on the task of classifying wild-type versus mutant morphological graphs. We split our data set 85%/15% train/test; all metrics we report are calculated on the test set. We compare the following four methods:

1. Original GDD of unweighted graphs, with t chosen as the arg⁡max of D over the values $t_{1}\ldots t_{k}$;
2. Weighted norm version of Diff2Dist, using graphs with Gaussian kernel edge weights (with σ fixed at $10^{-3}\cdot w_{max}$, where $w_{max}$ is the largest edge weight over all graphs in the training data set), with $t_{1}\ldots t_{k}$ and $\beta_{i}$ tuned via backpropagation;
3. Weighted norm version of Diff2Dist using graphs with Gaussian kernel edge weights, with t,σ and $\beta_{i}$ all tuned via backprop;
4. Weighted norm version Diff2Dist of graphs with general edge weights parameterized by a small neural network (see below for network specification). Input to this neural network was a vector of all three edge attributes.

For methods 2 and 3, the input to the distance kernel was the distance between nodes in the original image. All parameters were tuned using ADAMOpt (Kingma and Ba 2014) (with default PyTorch hyperparameters and batch size 256) to minimize the contrastive loss function (Hadsell *et al.* 2006):

(7) $$\begin{array}{rr} ℒ\left(G_{i},G_{j}\right) & \;=\frac{1}{2}(y_{ij}\text{max}{\left(0,D\left(G_{i},G_{j}\right)-\rho_{\text{lower }}\right)}^{2}\\ & +\left(1-y_{ij}\right)\text{max}{\left(0,\rho_{\text{upper }}-D\left(G_{i},G_{j}\right)\right)}^{2}) \end{array}.$$

This loss function encourages $G_{i}$ and $G_{j}$ to be closer than $\rho_{\text{lower }}$ if they have the same label, and further apart than $\rho_{\text{upper}}$ if they differ $\left(y_{ij}\right.$ is a binary indicator of label agreement). These margins were set $\left(\rho_{\text{lower}}=0.001,\rho_{\text{upper}}=0.33\right)$ by trial-and-error on the training set. Training took 600 epochs. For the neural network approach, edge weights were chosen as the final output of a neural network with seven layers of sizes {3,128,32,32,32,32,1} with sigmoid linear unit (SiLU) activations on the first six layers and no activation function on the last layer. We observed that the benefit of our approach was consistent across a variety of neural network architectures; we leave a more thorough evaluation of how network capacity influences this method for future work.

Results of these experiments are presented in Table 1. In this table, we compare the performance of a simple K-nearest-neighbour (KNN) classifier trained on the distance matrix produced by each variant method of calculating the distance. Reported values are the accuracy of the classifier on the test data set, and each KNN classifier uses the value of K that achieved the highest accuracy on the training data set. We see that the learned distance matrix, using neural network weights, yields a classifier with 95% accuracy on the test set. This observation demonstrates that our method is able to learn a distance metric that separates the two categories of graph more effectively than the diffusion distance metric alone.

We also compare our model to a baseline classifier: a neural net trained on per-graph histogram feature vectors. Sahlin *et al*. (2009) demonstrate that wild-type and mutant tissues in *Arabidopsis* are well-characterized by histograms of per-cell neighbour counts. We train a neural network (with SiLU activations and layer sizes [16,32,32,32,32,128,64,1]) to predict cell type from histograms of neighbour counts for each graph in each data set. On the SAM data set, this simple classifier outperforms regular GDD. However, our distance variants with learned components (approaches 2, 3 and 4) outperform this baseline classifier by a substantial margin. For the Replum data set, all four of our approaches outperform the baseline, but the full neural net enabled distance measure performs best (98% accuracy).

**Figure 4.**
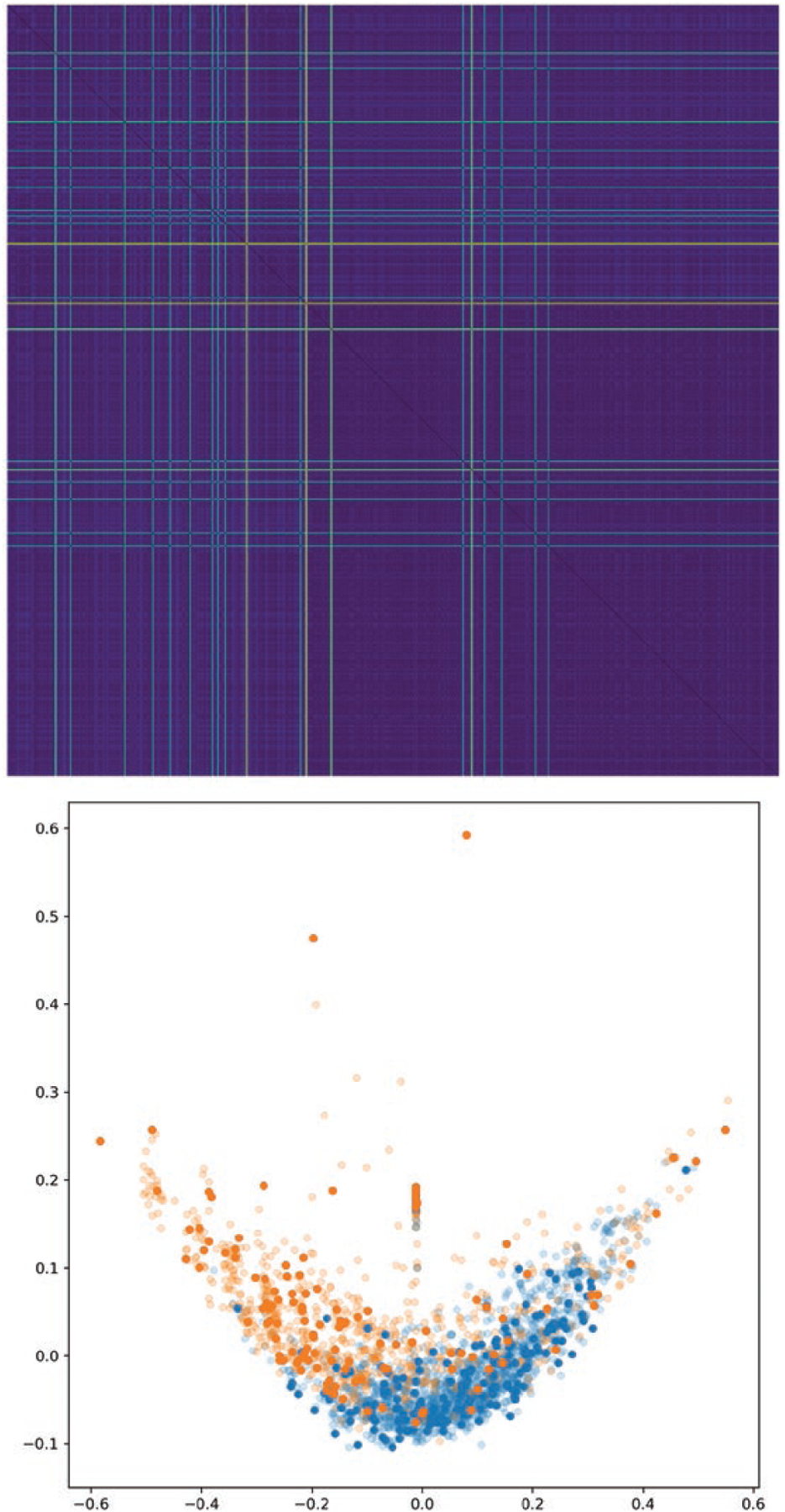
Top: distance matrix for morphological graphs, generated with method 1 (GDD on unweighted graphs). Bottom: Isomap embedding of this distance matrix, which ensures that points with small distance are placed near each other. Blue points represent the wild type, and orange points represent the mutant. Transparent points represent training set graphs; solid points represent those from the test set. We see that naive GDD leads to an embedding where the two categories of graph overlap, indicating that GDD by itself is not capturing the distinction between these two classes of graph.

**Figure 5.**
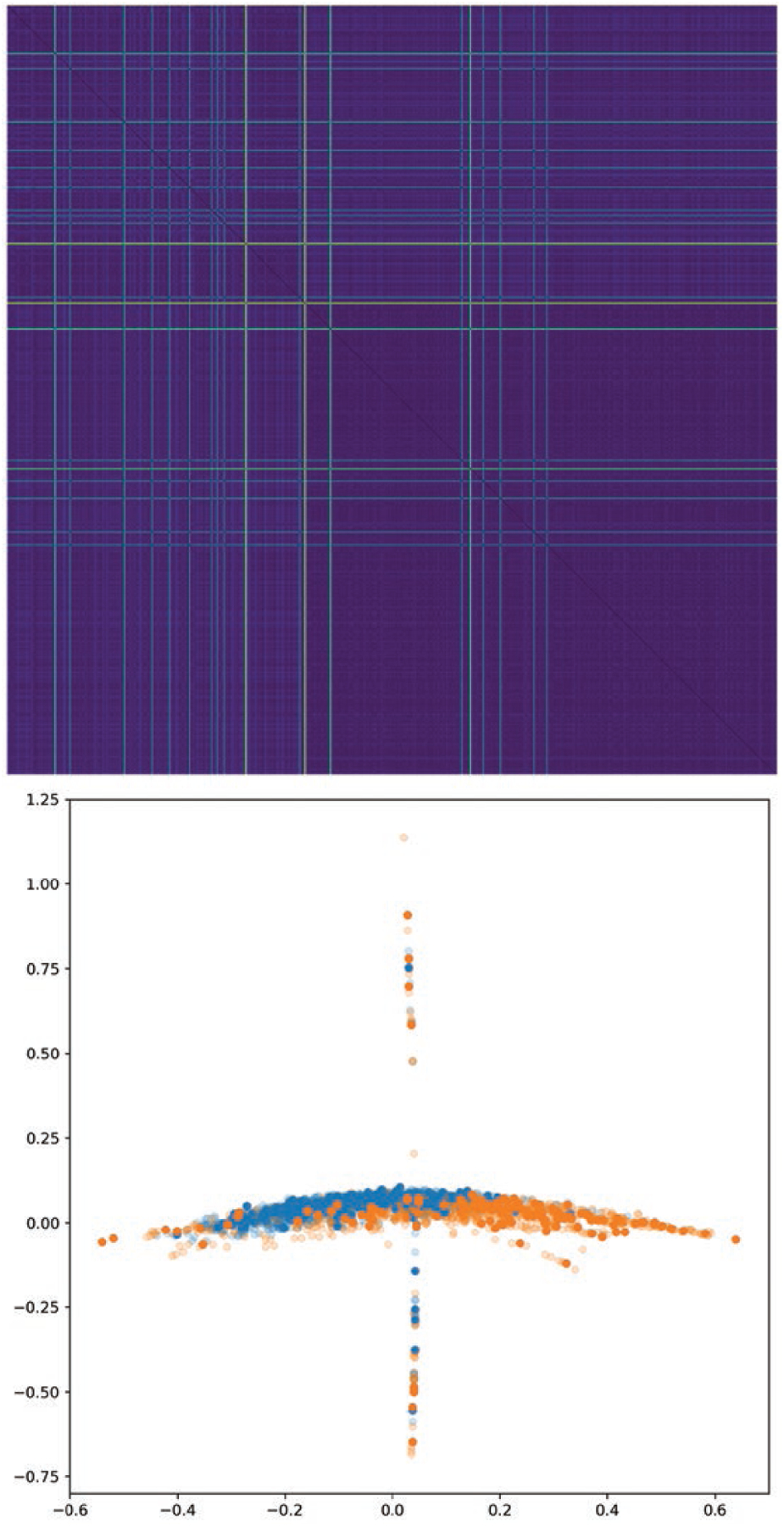
As in Fig. 4, but for method 2 (weighted norm GDD on graphs with fixed Gaussian kernel edge weights.). This distance matrix and embedding have the same flaws as those in Fig. 4.

**Figure 6.**
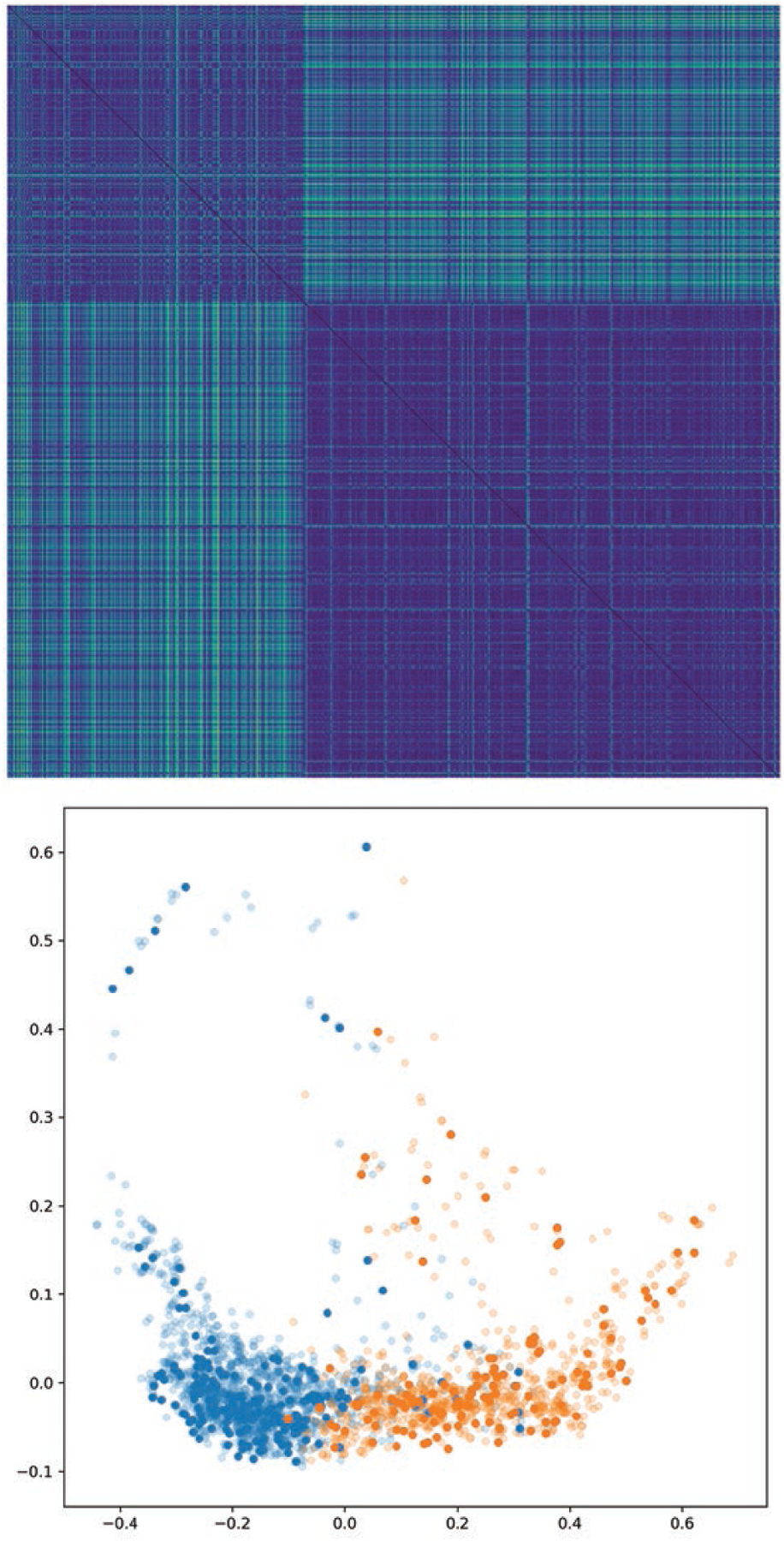
As in Fig. 4, but for method 3 (weighted norm GDD with Gaussian kernel weights with tuned). σ We see that using a Gaussian distance kernel for edge weights, with radius tuned by gradient descent, results in a graph distance metric which better separates the two categories.

**Figure 7.**
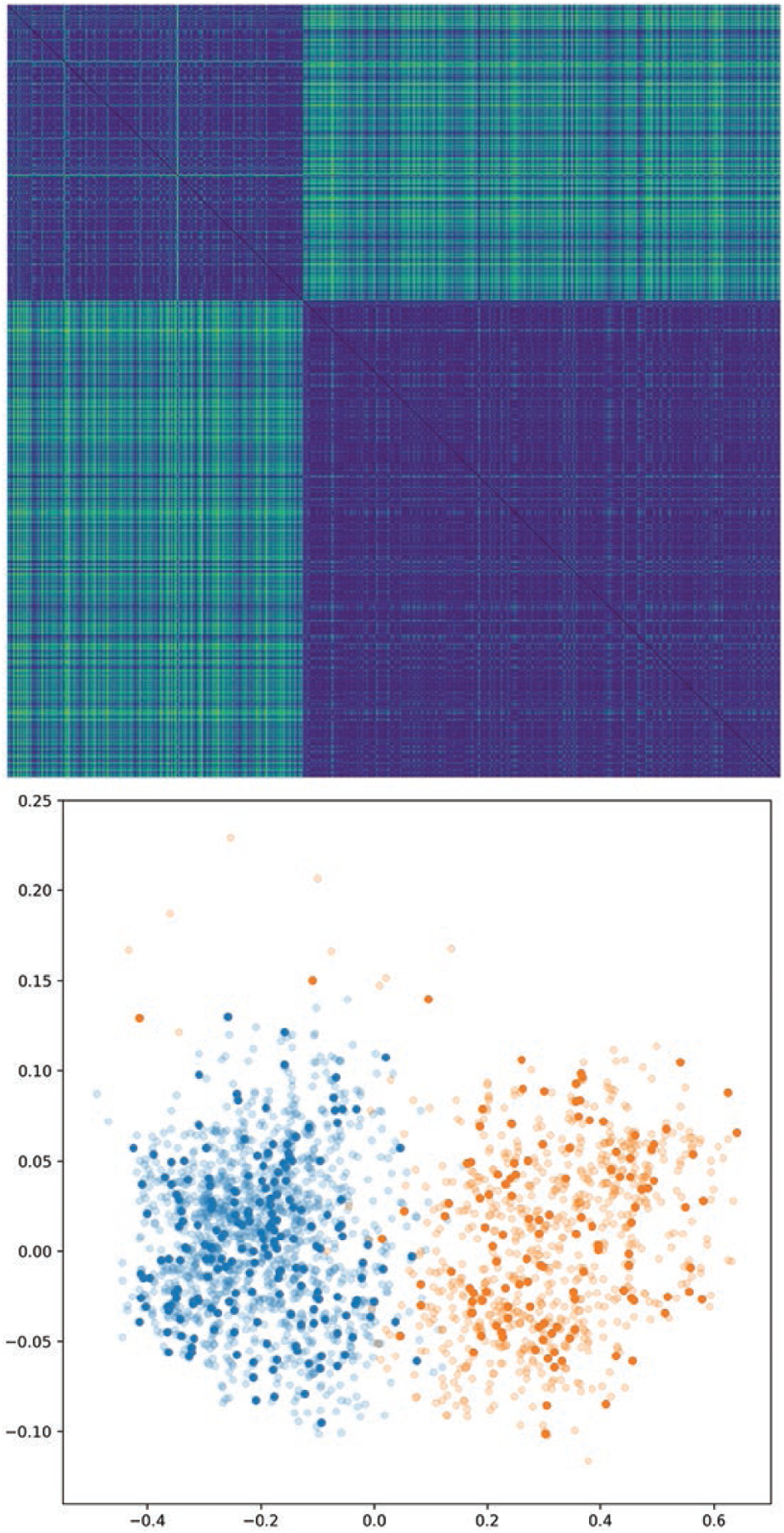
As in Fig. 4, but for the ANN-determined edge weights. Replacing the arbitrary Gaussian distance kernel with weights chosen by a machine learning model makes this approach fully general and produces a distance metric which fully separates the two graph categories. Blue points (left lobe): wild type. Orange points (right lobe): mutant.

We also present distance matrices for each approach on the SAM data set, as well as Isomap (Tenenbaum *et al.* 2000) embeddings of each (we used the scikit-learn (Pedregosa *et al.* 2011) implementation of Isomap with 15 neighbours and default hyperparameters). The distance matrices developed using the ANN approach clearly show better separation between the two categories. See Figs 4, 5, 6,7. In each of these figures we show the distance matrix for that graph distance method, as well as the result of embedding the distance using Isomap. In the Isomap embeddings, the points are coloured by cell type, with semitransparent points representing the training data set and opaque points representing the test data set. The distance matrices and resulting embeddings for methods 1 and 2 do not result in a clear separation between the two categories, whereas methods 3 and 4 do. The Isomap embeddings demonstrate that methods 1 and 2 all result in overlapping clusters, since some within-category distances are higher than some inter-category distances. In contrast, methods 3 and 4 (learning weights with a neural network model) both have two distinct clusters. Furthermore, method 4 (Diff2Dist) demonstrates a dramatic improvement over method 3, showing that this version of GDD can be tuned so that the resulting graph distance minimizes an arbitrary loss function (e.g. separates classes of graphs). This shows that our neural network is learning an edge weighting function which highlights some difference between morphological properties of the two classes of graphs. We note that these results are consistent across both of the *Arabidopsis* data sets considered in this paper.

## 5. ANALYSIS OF SIMULATION PARAMETERS

The previous section demonstrates that our modified version of GDD is able to reliably find structural features which distinguish wild type from mutant. In this section, we use the best trained model (the model with neural network chosen weights, referred to in the previous section as ‘approach 4’) as a tool to interpret which morphological features contribute the most to this discrimination. We use the distance metric trained in the previous section to characterize the differences in cell growth between the two types of cells, by comparing the biological graphs to graphs generated by a simulation that incorporates hypothesized mechanisms of growth control.

We generated a population of artificial morphological graphs using the simulation code ‘Tissue’ (Hamant *et* al. 2008). Tissue uses including finite element mechanical models to simulate growing collections of cells (represented as sets vertices). See the Tissue Gitlab (Jönsson 2018) for instructions on how to use this software. For all of these experiments, we started from the *meristem.init* file which is packaged with Tissue. In a typical Tissue simulation, cell walls act like springs with a fixed spring constant, and all cells grow isotropically at a fixed linear rate. Cell division can be triggered by cell size, shape, or randomly—we configured Tissue so that cells divide when they reach a volume of 40 arbitrary units, with a small random chance $r_{\text{div }}$ of dividing at any timestep if they are larger than 20 units (see the included configuration file for details). These numbers (40 and 20) were chosen so that the final mesh produced by each simulation had the same distribution of cell volumes as the data set mention in Section 4.1.1.

By default, Tissue places new cell division planes along the shortest path which divides the two daughter cells into roughly equal volume, following the well-known * Errera’s rule*(Errera 1886; Besson and Dumais 2011). The shortest path is found by enumerating each possible path and measuring their lengths. We modified this behaviour so that, with probability $r_{\text{angle, }}$ one of the less optimal paths was chosen instead. These parameters were implemented in a new division rule we contributed to the Tissue simulator (our modified version of Tissue’s source code is available in Code Repository of this manuscript). We also varied two parameters from the original version of Tissue, representing the spring constant of each cell wall and the exclusion radius around each vertex (where new vertices are not allowed to be placed during division). A summary of the values swept over for each parameter is in Table 2. There were 4×3×4×6=288 combinations of parameters, resulting in that many simulations.

Each simulation resulted in a mesh file representing the final positions of cell vertices and cell walls after 10 000 timesteps. Each mesh was converted into an image and processed into a set of graphs (one centred on each cell) exactly as described in Section 4.1.1. This yielded a large secondary data set of synthetically constructed morphological graphs.

We used the Diff2Dist model (trained on the biological graphs only) to compute the distance between each biological graph and all of the graphs which originated from a simulation. Each biological graph can then be assigned a numerical label for each parameter, by taking the mean of the parameters for the 100 closest simulations. This gives us an estimate of which parameter values a given biological graph is most similar to (under our learned distance estimate, which is shown to separate * trm678* and wild-type graphs).

**Figure 8.**
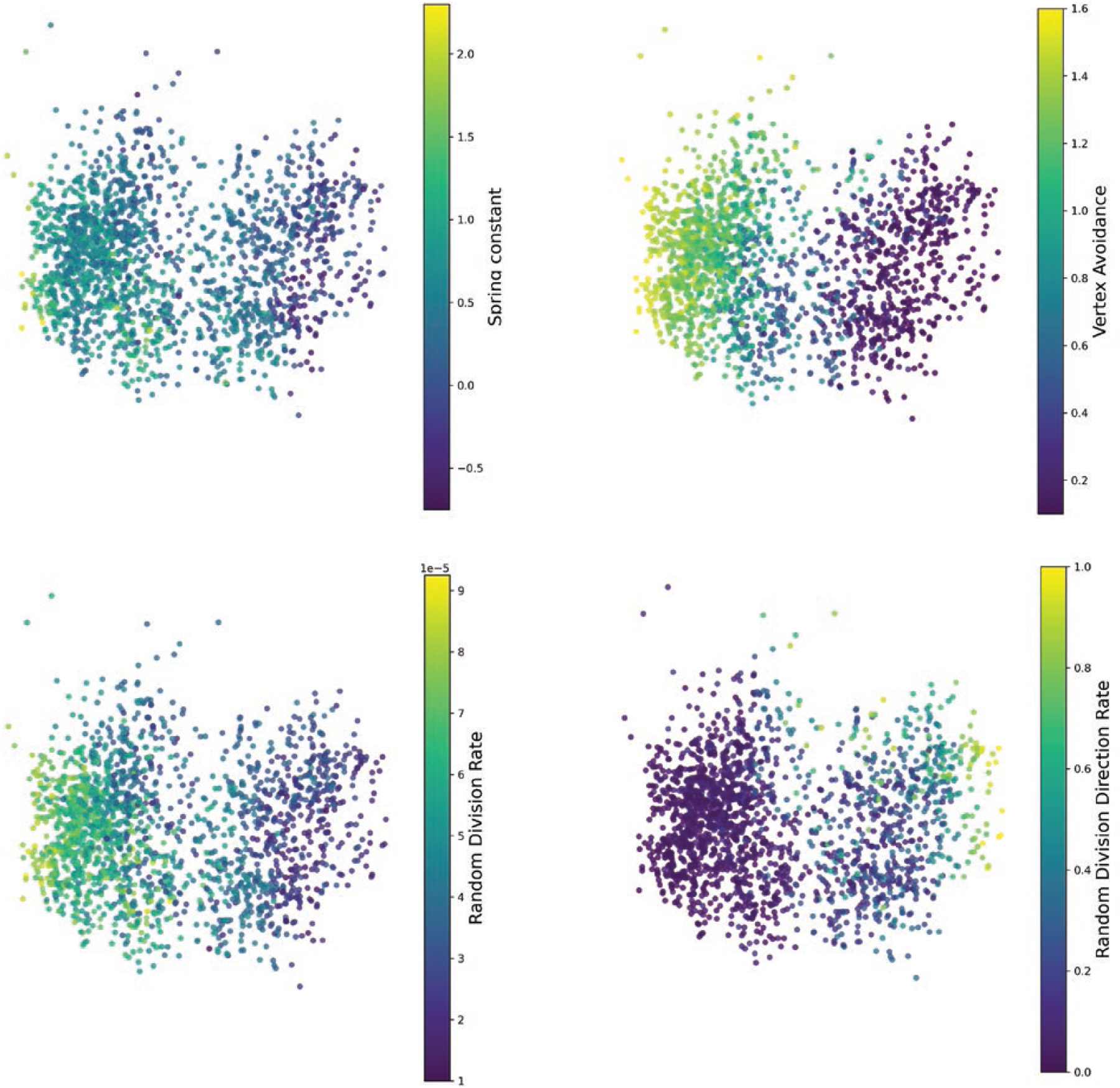
Visualizing simulation parameters using Diff2Dist. Each plot shows one point for each morphological graph in our *Arabidopsis* data set. Points are placed in 2D using Isomap, exactly as in Fig. 7. Points are coloured according to the parameter values of the nearest simulationderived graphs, where means in the sense of our trained distance metric.

The results of this experiment can be seen in Fig. 8. Comparing biologically derived graphs to synthetically generated ones in this way allows us to see that wild-type graphs are characterized by higher vertex avoidance (upper right panel) and lower rate of random cell division direction (lower right panel), whereas* trm678* graphs are more likely to have cell divisions in random directions.

## 6. CODE REPOSITORY

Code for all of the experiments in this paper is available as an Open Science Framework repository at https://osf.io/h2fzp/?view_only=02bf54a68eef469baa68721430c8c77f.

## 7. CONCLUSION AND FUTURE WORK

This paper presents a method to compute distance metrics between edge-labelled graphs, in such a way as to respect class labels. This approach is flexible and can be implemented entirely in PyTorch, making it possible to automatically differentiate the distance computation and thereby learn a distance metric between graphs that were previously not able to be discriminated by GDD. We demonstrate that our learned distance metric both (i) enables more accurate classification of cell types, and also (ii) allows us to use simulated data to interrogate the trained distance model, to determine what parameters characterize different types. We also demonstrate that our learned distance metric and associated classifier outperform previous classifiers based on topological features, indicating that geometric information is necessary to distinguish cell type.

In the future we hope to apply this method to more heterogeneous graph data sets by including the varying-size version of GDD. Additionally, we aim to apply this approach to data sets representing 3D cell topologies and data from more perturbed mutant plants. Our neural network approach, as described, is not a Graph Neural Network in the sense described by prior works such as (Kipf and Welling 2016; Bacciu *et al*. 2020), as there is no message-passing step. We expect message-passing layers to directly improve these results and hope to include them in a future version of differentiable GDD.

## Figures and Tables

**Table 1.** Validation set accuracy for a simple KNN classifier for all four methods, as well as a simple classifier as baseline. The test set was the same for each of these tests. For KNN-based methods, the value reported is the highest value of accuracy over all K in the range [3..50].

Method	Accuracy % (SAM cells)	Accuracy % (replum cells)
Histogram classifier	79.4	79.1
GDD only	75.6	85.1
*t*-tuning and *β*-weights	82.2	86.5
*t* and σ-tuning, *β*-weights	90.0	87.8
ANN parameterization	**97.2**	**98.6**

Bold values indicate the best-performing model on each dataset.

**Table 2.** Summary of input parameters and values used during comparison of simulations to biologically derived graphs. Spring constant controls the stiffness of cell walls during the simulation. Vertex exclusion size is the proportional size of an envelop around each vertex where new vertices may not be placed during division. $r_{\text{div }}$ is the random chance of a cell dividing at a given timestep even when it has not reached 40 units of volume. $r_{\text{angle }}$ is the probability (given that division has occurred) that the placement of the new cell wall will be random instead of optimal.

Parameter name	Values used
Spring constant	0.1, 0.3, 1.0, 3.0
Vertex exclusion size	0.1, 0.3, 0.6
Random division frequency *r*_div_	0.0, 0.00001, 0.00003, 0.0001
Random division direction frequency	0.0, 0.01, 0.03, 0.1, 0.5, 1.0
*r*_angle_	
